# Diabetes and Exposure to Environmental Lead (Pb)

**DOI:** 10.3390/toxics6030054

**Published:** 2018-09-06

**Authors:** Todd Leff, Paul Stemmer, Jannifer Tyrrell, Ruta Jog

**Affiliations:** 1 Institute of Environmental Health Sciences, Wayne State University, Detroit, MI 48202, USA; pmstemmer@wayne.edu; 2 Department of Pathology, Wayne State University, Detroit, MI 48202, USA; jannifermcintosh@gmail.com (J.T.); fj1102@wayne.edu (R.J.); 3 Center for Integrative Metabolic and Endocrine Research, Wayne State University, Detroit, MI 48202, USA; 4 Department of Pharmacology, Wayne State University, Detroit, MI 48202, USA

**Keywords:** lead (Pb), diabetes, rodent models

## Abstract

Although the increased incidence of type 2 diabetes since the 1950s is thought to be primarily due to coincident alterations in lifestyle factors, another potential contributing factor in industrialized countries is exposure of the population to environmental pollutants and industrial chemicals. Exposure levels of many environmental toxicants have risen in the same time-frame as the disease incidence. Of particular interest in this regard is the metal lead. Although overall lead exposure levels have diminished in recent decades, there is an under-recognized but persistent occurrence of lead exposure in poor underserved urban populations. Although the neural developmental pathologies induced by lead exposures have been well documented, very little is known about the effect of lead exposure on the incidence of chronic metabolic diseases such as type 2 diabetes. Although our understanding of the metabolic health effects of lead exposure is incomplete, there are studies in model systems and a small amount of epidemiological data that together suggest a deleterious effect of environmental lead exposure on metabolic health. This article reviews the human, animal and in vitro studies that have examined the effects of lead exposure on the development of diabetes and related metabolic conditions.

## 1. Introduction

The well-documented dramatic increase in the incidence of type 2 diabetes that has occurred since the 1950s is thought to be primarily due to coincident alterations in general lifestyle factors in affected populations. Chief among these are increased caloric intake, altered nutrient quality, and a general reduction in physical activity. Another potential contributing factor in industrialized countries is exposure of the population to environmental pollutants and industrial chemicals. Exposure levels of many environmental toxicants have risen in the same time-frame as the disease incidence. In addition, some of these widely distributed chemicals have been shown to interact with biological systems and to result in deleterious metabolic and/or endocrine effects. There is increasing evidence that exposure to certain of these endocrine disrupting chemicals (EDCs) alters diabetes risk [1].

One such EDC is the synthetic organic compound bisphenol A (BPA), which has been used in plastics manufacturing since the 1950s. BPA has been shown to interact directly with members of the estrogen receptor family, and importantly has been detected in the urine of greater than 95% of the US population [2]. Although there are conflicting views about the health effects of BPA exposure, the wide distribution of this chemical in the human population coupled with its ability to alter the activity of important endocrine pathways, illustrates the potential danger posed by the presence of environmental pollutants in the human habitat.

An important class of environmental chemicals that pose a human health risk are the metals with no known role in normal human physiology. Although many metals are required for normal biological functioning (e.g., zinc, cobalt, iron, copper, manganese, chromium, and molybdenum), a subset of metals have no role in animal physiology and can be considered xenobiotic—e.g., lead, mercury, cadmium and the metalloid arsenic. These elements have negative effects on physiology and in some cases human exposure levels have been associated with the incidence of diabetes and related metabolic syndromes [3,4,5,6,7,8,9,10,11].

Of particular interest in this regard is lead. Although overall lead exposure levels have diminished in recent decades, there is an under-recognized but persistent occurrence of lead exposure in certain sectors of the population. In particular, individuals living in poor urban communities with older housing stock are at significant risk for childhood lead contamination. In addition, the recent incident in Flint Michigan, where a large portion of the population was exposed to elevated lead in the municipal drinking water system [12], has raised awareness of a potential drinking water route of exposure that could put larger segments of the population at risk of lead exposure.

Between childhood exposures from ingestion of lead-based paint and sporadic exposure of adults through contaminated drinking water, there probably remains a significant segment of the population exposed to low levels of lead. Although the neural developmental pathologies induced by lead exposures have been well documented [13,14,15], very little is known about the effect of lead exposure on the incidence of chronic metabolic diseases such as type 2 diabetes.

The question of whether low levels of lead exposure can increase susceptibility to diabetes must be considered in light of the fact that the most exposed populations are often encumbered with additional metabolic, nutritional and environmental stressors. For example, underserved urban populations have higher rates of obesity and are generally exposed to higher levels of socio-economic and environmental stress than the general population [16]. If lead exposure does have a pro-diabetic effect on physiological systems, the clinical outcome of exposure may be more severe in these underserved urban populations than observed elsewhere.

Although our understanding of the metabolic health effects of lead exposure is incomplete, there are studies in model systems and a relatively small amount of epidemiological data that together suggest a negative effect of environmental lead exposure on metabolic health. In this article we will review the animal and in vitro studies that pertain to this issue as well as studies on the epigenetic effects of lead exposure that address the issue of how pediatric exposure to lead might have long-term influences on general health later in life.

## 2. Human Lead Exposure Patterns

In the US population lead exposure, as measured by blood lead levels, has been declining for several decades. This is due in large part to the removal of lead from gasoline in the 1980s, which is regarded by many as one of the major public health triumphs of the 20th century. This change did indeed have a rapid effect on the overall level of lead exposure in children, which saw a drop in the mean blood lead concentration from 13.7 µg/dL to 3.2 µg/dL between 1976 and 1994. This trend has continued, and the CDC estimated the average blood lead level in 2005 had diminished to 1.5 µg/dL [17]. It is easy to understand how this truly dramatic improvement in the overall level of lead exposure in the general population has engendered a belief that the lead toxicity problem has been solved.

There are several important observations however, that run counter to the view that lead toxicity is no longer a health issue. First, examination of the rates of childhood blood lead exposures between 1997 and 2015 [18] reveals a persistent fraction of children (about 0.5% of those tested) diagnosed with significantly elevated blood lead (<10 µg/dL). The CDC estimates that there are currently about 500,000 children ages 1–5 in the US with blood lead levels above 5 µg/dL, the reference level at which CDC currently recommends public health actions be initiated [19]. Furthermore, when one considers the fact that the current, seemingly low, average blood lead levels in the US are still many thousand-fold higher than estimated prehistoric levels [20], and the fact that even low concentrations of lead can affect the function of many biological systems (summarized below), it is clear that exposure to environmental lead is still a significant public health problem.

An important characteristic of the current landscape of lead exposure in the US is its non-uniform distribution. Exposure levels are higher in poor, under-served populations mainly in urban industrial areas. Since the removal of leaded gasoline, most recognized lead-exposures occur in one of two scenarios: ingestion of lead-based house paint (in the form of lead-contaminated household dust) by toddlers, and the consumption of lead-contaminated drinking water. The former occurs in urban areas where the housing stock has not been modernized since the discontinued use of lead paint in the 1970s [21], and the latter in areas where the municipal infrastructure systems have not been properly maintained.

Although the circumstances leading to pockets of elevated lead exposure occur throughout the country, typical examples can be found in the state of Michigan. In the city of Detroit there are neighborhoods where nearly half of children tested had blood lead levels above 5 µg/dL as recently as 2006 [21]. These exposures are most likely due to the prevalence of houses containing lead-based paint in these neighborhoods. Recent events in the nearby city of Flint Michigan illustrate another route of human lead exposure that may be more common than generally appreciated. In 2014, a change in the quality of the city’s municipal water source resulted in a solubilization of lead from lead-based pipes in the city’s water distribution system. This increased the concentration of lead in drinking water and promoted the development of elevated blood lead levels [12]. In the highly publicized aftermath it became apparent that many municipal water systems across the country were at risk of releasing lead into the water and putting large segments of the population at risk of lead exposure.

In conclusion, while there have been dramatic reductions in the severity of lead exposure in the general populations there is still a significant level of exposure in many segments of the population.

## 3. Evidence that Lead Exposure is Pro-diabetic 

### 3.1. Association of Lead and Other Metals with Metabolic Disorders

Although there is substantial epidemiological evidence that exposure to low levels of various environmental chemicals can influence the development of chronic metabolic diseases including diabetes [22], there are very few studies specifically designed to examine the effect of lead-exposure on diabetes development. In the absence of such studies, it may be informative to examine investigations on the metabolic effects of other metals. Additional relevant information can be derived from epidemiological studies on lead exposure that while not directly measuring diabetes risk, have examined pathologies related to diabetes (e.g., fatty liver) that can be interpreted as indirect markers of the disease. This section will review these studies as well as the small number of studies that have looked specifically at lead exposure levels and diabetes incidence.

As mentioned above, only a small number of studies have directly examined the correlation between blood lead levels and diabetes incidence. Kolachi et al. found that blood hair and urine lead was higher in diabetic compared to non-diabetic females. However, both cadmium and arsenic were higher in the diabetes group, making it difficult to assign a specific role to lead [11]. In an earlier study with factory workers in the United Arab Emirates significant positive correlations between blood lead levels and fasting blood glucose were observed, suggesting a possible link between lead exposure and diabetes. This study also found an association between lead exposure and blood pressure [10].

Numerous studies have examined the effects of metal exposures on the function of the endocrine pancreas [3]. Although lead levels were not independently evaluated in these studies, together they suggest a deleterious effect of metal exposure on the islet function and are consistent with the possibility that lead exposure degrades endocrine pancreas function.

Fatty liver, especially the form not associated with excessive alcohol consumption—known as NAFLD (non-alcoholic fatty liver disease) co-exists with type 2 diabetes. In some populations up to 70% of diabetic patients also have NAFLD [23,24], which makes NAFLD incidence a reasonable predictor of type 2 diabetes rates. Given the physiological relationship between these two syndromes, an observed correlation between fatty liver and lead exposure could suggest a causative link between exposure to lead and diabetes.

In a recent study of the population living in the Yangtze river delta in China, Zhai et al. found that elevated blood lead levels were associated with an increased risk of NAFLD in both men and women, although the association was significantly stronger in women [5]. Consistent with this NAFLD study is an earlier study carried out with data from the 2003–2004 NHANES cohort. Cave et al. observed correlation between blood lead levels and a general marker of liver disease—elevated serum alanine aminotransferase (ALT) [4]. Although altered ALT levels are associated with multiple types of hepatic dysfunction, the observation that they are elevated in lead-exposed individuals is consistent with there being a causative link between lead exposure levels and the kinds of liver dysfunction associated with diabetes, such as NAFLD. It must be noted however that in this study lead levels were evaluated together with the heavy metals mercury and cadmium, so it is not possible to assign the deleterious liver effects specifically to lead exposure.

Although, as discussed above, there are few studies that have specifically examined the effect of lead exposure on diabetes rates in the human population, several studies have looked for correlations between lead exposure and other metabolic and endocrine parameters that are potentially related to diabetes.

Many of these studies examined industrial lead workers who had substantially higher levels of exposure than typically seen today (see above). Although this may reduce the direct relevance of these studies to general human exposure situation, they highlight potential physiological effects of lead that may be minor when exposure levels are low, but that still may pose a long-term health threat as the exposed population ages. Studies examining lead industry workers identified a mild correlation of blood lead levels with systolic blood pressure [25] and kidney function [26,27,28].

The findings from highly exposed individual are consistent with observations from non-industrial worker cohorts where correlations between lead levels and kidney function were observed even at much lower levels of exposure. Using data from the normative aging study Tsaih et al. observed a correlation between specific parameters of kidney function and lead levels in blood and bone [29], and that this effect was stronger in patients with diabetes and hypertension. A similar effect on kidney was seen in a study on teenagers where higher blood lead levels correlated with reduced kidney function (glomerular filtration rate) [30].

While these findings do not directly address the question of whether lead-exposure in the human population promotes the development of diabetes, they do demonstrate that lead has deleterious effects on physiological systems that are crucial for the maintenance of normal metabolic balance.

### 3.2. Rodent Studies

Although there have been more studies in animal models than in humans, there is still a relatively small literature on the effect of lead exposure on metabolism in animal models. Several early studies in the 1970s and 80s suggested that lead exposure could induce hepatic insulin resistance in rats. Singhal et al. [31] reported that male rats treated with lead acetate by intraperitoneal injection, showed a dose- dependent increase in amounts of the gluconeogenic enzymes phophoenolpyruvate carboxykinase (PEPCK) and glucose-6-phosphatase (G6Pase) in the liver. Consistent with the lead-induced increases in gluconeogenic gene expression, this study also reported a dose-responsive increase in fasting blood glucose. A similar finding was reported by Stevenson et al. in young female rats exposed to 20, 40 or 80 ppm lead for 56 days in drinking water [32]. These animals also showed a dose-responsive increase in PEPCK and G6Pase enzyme levels in the liver and significantly elevated fasting blood glucose. Finally, Whittle et al. demonstrated that even small increases in blood lead levels from a control level of 7.9 ± 1.4 µg/dL to 13.8 ± 1.3 μg/dL, for 42 days caused a significant increase in expression of hepatic gluconeogenic enzymes in rats [33].

While these findings strongly suggest that lead exposure induces hepatic glucose production and thereby contribute to elevated blood glucose levels, they were somewhat limited in technical scope and do not provide much information about potential mechanisms by which lead has these effects in the liver.

The pro-diabetic effects of lead exposure on the liver were observed in recent studies that examined the effect of lead on hepatic metabolism in normal rats. When rats were exposed to lead acetate in drinking water, increased enzymatic activity of hepatic PEPCK and G6Pase were observed together with a mild increase in fasting glucose and glucose intolerance [34]. Interestingly, in obese rats the metabolic effects appeared to be stronger with pronounced elevated glycemia and glucose intolerance [35]. Both studies also explored potential mechanism by which lead might affect metabolic balance. Mostafalou et al. demonstrated that ex vivo, lead treatment suppressed glucose stimulated insulin secretion from islets, possibly by activating glycogen synthase kinase (GSK-3β kinase) [34]. Tyrrell et al. demonstrated that livers from exposed rats had elevated transcript levels of both PEPCK and G6Pase gluconeogenic genes and also showed that this effect could be recapitulated in vitro in lead-treated hepatoma cell lines [35]. Together these studies indicate that lead can have direct effects on cells from metabolically relevant organs.

### 3.3. Epigenetic Effects of Lead Exposure

As described above, a common route of lead exposure in humans occurs early in childhood by ingestion of lead-contaminated household dust [36]. This exposure route is generally limited to early childhood during the period in which toddlers ingest large amounts of household dust. As children age they dramatically reduce the ingestion of non-food material and their exposure to lead diminishes. This childhood exposure pattern raises the question of whether a limited exposure to lead early in life could induce persistent changes in physiology, even in the absence of continued exposure, that manifest themselves as disease later in life.

A substantial literature exists related to how early in life conditions influence later in life health; essentially the DOHaD or Developmental Origins of Health and Disease hypothesis [37]. There are multiple examples of specific early in life stresses—for example nutritional deprivation [38], emotional stress [39] or chemical exposures [40]—that have negative effects on various aspects of adult health. These long-lasting health effects of early life stresses, can be attributed to specific epigenetic mechanisms such as altered DNA methylation patterns [37]. Previous studies have shown that exposure to environmental chemicals can induce epigenetic changes that persist for long periods of time and potentially affect physiology and health [41]. In the context of childhood lead exposure in humans and its potential adult health effects, it is of interest to know if lead exposures induce detectable epigenetic changes of any variety.

There is clear evidence that lead exposure induces changes in DNA methylation at specific loci in humans. Sen et al. examined umbilical cord blood from the Early Life Exposure in Mexico to Environmental Toxicants (ELEMENT) study participants [42]. When cord blood from individuals in the lowest and highest lead exposure groups were compared, clear differences in DNA methylation patterns were observed. Analogous findings were also observed in populations of Detroit mothers and infants [43]. These findings raise several interesting possibilities. First, it is possible that specific DNA methylation patterns might be useful as early biomarkers of prenatal lead exposure. Second, they raise the possibility that early life lead exposures leave long lasting epigenetic imprints that alter physiology and potentially affect health later in life. 

An interesting aspect of epigenetic imprinting is the potential for inheritance of imprints across multiple generations. With regard to lead exposure, Sen et al. found that moms with high blood lead had children as well as grandchildren with exposure-specific epigenetic DNA methylation patterns [43]. If DNA methylation patterns do indeed have the capacity to influence physiology, then these findings raise the possibility that lead exposure in a previous generation could have effects on health in subsequent generations.

The possibility that early life lead exposure induces later in life health problems is supported by multiple studies in rodents. When mice were perinatally exposed to levels of lead similar to those commonly seen in humans, they exhibited neurological deficits in adult mice and late-onset obesity [44]. In another study perinatal lead exposure was found to affect multiple metabolic and activity parameters over the course of the life of the animal [45]. Interestingly, these authors observed sex differences in how lead exposure affected metabolic parameters. Finally, analogous to the human studies described above, persistent DNA methylation patterns at specific loci were induced by perinatal-limited exposure to lead [45,46].

## 4. Molecular Mechanisms by Which Lead Might Promote Diabetes

### 4.1. Lead Causes Oxidative Stress, a Risk Factor for Diabetes

If lead exposure does promote the development of diabetes as suggested by the above animal studies, what might be the molecular basis for this effect? One possible mechanism is based on the well-known effect of lead to induce oxidative stress in biological systems [47]. This is relevant as several key components of the insulin signaling pathway are known to be inhibited by reactive oxygen specific (ROS), promoting the development of insulin resistance and diabetes [48]. Recent studies revealed a strong association of blood lead with markers of oxidative stress in the general population and suggested that oxidative stress should be considered in the development of lead-mediated diseases, even among people with relatively low environmental exposure to lead (i.e., <10 μg/dL) [49].

Unlike redox active metals such as iron and copper, Pb^++^ does not change its valence state and cannot generate ROS by the Fenton reaction. However, it is clear that Pb^++^ increases oxidative pressure in living organisms [50,51,52], and this takes place by four proposed mechanisms each of which is supported by experimental evidence. First, Pb^++^ changes the lipid composition of membranes, resulting in a decrease in phosphatidyl choline and an increase in arachidonic acid [53,54]. The change in lipid composition is coupled to increased susceptibility to lipid peroxidation (e.g., an increase in malonyldialdahyde is found in the presence Pb^++^) [53,54].

Second, Pb^++^ stimulates superoxide formation by binding to oxyhemoglobin [55,56,57]. Third, the enzyme porphobilinogen synthase [aminolaevulinate dehydratase; ALAD], involved in heme biosynthesis, has extreme sensitivity to Pb^++^ ions. Lead has a direct effect on ALAD via active-site inhibition. Specifically, Pb^++^ binding displaces an essential Zn^++^ ion at the catalytic site and therefore inhibits ALAD activity [58,59]. Consequently, δ-aminolevulinic acid (ALA) becomes elevated in blood and could potentially generate superoxide through an autoxidation mechanism [60,61,62]. Elevated levels of ALA are present in the blood and urine of lead-exposed individuals. Finally, Pb^++^ decreases super oxide dismutase (SOD) and glutathione peroxidase (GPx) antioxidant activities by binding to sulfhydryl groups or selenocysteine, which are required for their antioxidant activities [63,64,65].

Oxidative stress is thought to promote the diabetic state by direct effects on cellular signaling pathways that influence insulin signaling. One proposed mechanism is that ROS activate intracellular signaling through JNK/SAPK, p38 MAPK and NF-κB. JNK activation results in serine phosphorylation and inhibition of IRS (Insulin Receptor Substrate) 1 and 2. IRS1 and IRS2 are required for downstream signaling through additional serine/threonine kinases and their phosphorylation by JNK results in decreased insulin signaling and insulin resistance [48,66].

Pancreatic beta-cells appear to be exquisitely sensitive to reactive oxygen species (ROS) [67]. The heightened sensitivity of beta-cells in comparison with other cell types is attributed to an unusually low level of antioxidant enzymes, including SOD, catalase, and GPx [67,68,69]. The deficit in enzymatic antioxidant capacity can be overcome by expression of exogenous genes for the antioxidant enzymes [70] or by providing antioxidant drugs [70,71]. Several antioxidants, including lipoic acid, N-acetyl cysteine and vitamin C, reduce insulin resistance in type 2 diabetes [70,71]. It remains to be determined if antioxidant therapy can prevent or slow the development of type 2 diabetes and to identify the signaling pathways that can be modulated to decrease the amount of oxidative stress in pancreatic islets.

### 4.2. Lead Alters Intracellular Signaling Pathways

#### 4.2.1. Lead Increases Resting Intracellular Ca^++^

Pb^++^ and Ca^++^ have very similar electron orbitals. Most of the effects of toxicity are a result of this similarity in their molecular features. Thus Pb^++^ can interfere with a variety of intracellular processes where normally Ca^++^ would have been involved. For example, Pb^++^ interferes with calcium homeostasis and cellular uptake by binding at the same binding sites as Ca^++^ on different Ca^++^-binding channel pore proteins and transporters. At pM concentrations, Pb^++^ increases resting intracellular Ca^++^ concentration from approximately 100 nM to 200 nM [72]. Although the consequence of a Pb^++^-dependent increase in intracellular Ca^++^ concentration has not been well studied, it undoubtedly exerts a significant effect on modulating calmodulin (CaM)-dependent events, e.g., activation of calcineurin. Because oxidants are effective calcineurin inhibitors only when the enzyme is active [73], a persistent activation of calcineurin by an increased resting Ca^++^ concentration could produce a sustained oxidative inactivation of calcineurin.

With effects of Pb^++^ on calcineurin in the low nM range it is likely that lead has a direct effect on calcineurin action and thereby could influence calcineurin-dependent cellular activities in for example, insulin-producing pancreatic β-cells [74]. Kern and Audesirk [75] have shown that calcineurin is stimulated by pM levels of Pb^++^.

Most findings reported in the literature indicate that direct binding of Pb^++^ by CaM does not contribute to the toxic effects of Pb^++^ in intact cells [76,77,78]. Even if there is positive co-operativity and target-dependent increases in affinity for Pb^++^ binding to CaM to the same degree as occurs with Ca^++^, the intracellular Pb^++^ concentration never reaches the high nM range required to activate CaM-dependent enzymes in vitro [76,77,78,79]. This does not rule out an *indirect* effect of Pb^++^ on CaM-dependent signaling. There are potentially two mutually reinforcing mechanisms by which CaM binding to reservoir proteins such as neuromodulin and neurogranin, and other targets might be modified by Pb^++^. First, because CaM reservoir proteins are phosphorylated by Pb^++^ stimulated PKC [80,81,82], CaM is inhibited from binding to them, which reduces the available CaM concentration in localized cellular compartments. Second, as a result of increased intracellular Ca^++^ concentration, the affinity of reservoir proteins for CaM is decreased and CaM redistributes away from reservoir locations [83,84,85,86].

#### 4.2.2. Lead Modulates PKC Activity

Lead activates PKC at low concentrations and inhibits it at higher concentrations [87]. At pM concentrations, Pb^++^ activates PKC in adrenal chromaffin cells [88] and whole-brain extracts [89,90]. In vivo, lead nitrate upregulates PKC-ε and downregulates PKC α and β [91], results that are consistent with the variable findings of PKC inhibition in astrocytes and vascular cells at micromolar Pb^++^ concentrations. Inhibition of PKC by Pb^++^ at micromolar concentrations has been associated with effects on neurotransmitter and c-Fos activity in PC12 cells [92]. However, the sequelae of stimulation (or inhibition) by Pb^++^ at pM concentrations have not been defined in any cell type. The chronic nature of Pb^++^ exposure makes any effect mediated through PKC more complex because PKC responds to stimulation by downregulating activity [93,94]. Therefore, lead might initially stimulate PKC activity, which might decrease its sensitivity to normal stimuli.

#### 4.2.3. A Potential Link Between Lead and Rev-erb-α

Recent studies indicate that components of the circadian clock machinery regulate expression of metabolic genes, including PEPCK, G6Pase and PGC1-α [95]. This evidence was further supported by data gathered from mice with mutated Clock genes showing abnormal gluconeogenic gene expression [96,97,98]. One of the transcription factors that mediates circadian regulation of metabolic genes is the orphan nuclear receptor Rev-erb-α (NR1D1) [99]. Recently it has been demonstrated that heme functions as an endogenous ligand for Rev-erb-α and is required for its transcriptional repressive activity in the liver [100,101,102,103]. Heme binding to Rev-erb-α suppresses the transcription of Rev-erb-α target genes including PEPCK, G6Pase and PGC1-α [101,102]. Thus, in the absence of heme, the gluconeogenic program is elevated. The well described effect of lead in the inhibition of heme biosynthetic enzymes [104,105] (described above) raises the possibility that lead exposure leads to elevated gluconeogenesis by reducing the suppressive effect of Rev-erb-α on gluconeogenic gene expression.

## 5. Conclusions/Future Prospects

It is tempting to view the coincidence of the relatively high levels of lead exposure of the last century with the rise in diabetes rates that have occurred in this century, even if there is little epidemiological evidence to support this. Although relatively limited in scope and conducted at higher levels of blood lead than typically seen in human exposures (14–74 µg/dL [33,34] compared to the human alarm level of 5 µg/dL) the small number of animal studies that have been carried out support the idea that at some level of exposure, perhaps in combination with other metabolic stresses, lead promotes the development of diabetes. When one considers that even very low doses of lead are likely to have harmful effects [106], and that there remains a significant segment of the population that is exposed to environmental lead, it is clear that the effects of lead exposure on the metabolic health of the population remains an important research topic.

The possibility that early in life lead exposure might have later in life physiological effects is supported by the observations in humans and mice that lead exposure leads to specific epigenetic imprints in the genome that persist throughout life and even across generations. Although it is not yet possible to directly link specific patterns of epigenetic imprinting to specific physiological or pathophysiological parameters, it is likely that such links exist. This may ultimately provide a clear mechanistic pathway for how a limited exposure to lead might have long-lasting health effects.

Although the solution to the public health problem is clear-—if lead is removed from the built environment the risk of exposure falls-—it is clearly still worthwhile to understand the mechanisms by which lead affects biological systems, and to determine the exact contribution that lead exposures play in determining diabetes risk. For one thing, it is unlikely that the risk of exposure to environmental lead will be removed in the near future. Human exposures, even if at a low level are likely to continue into the foreseeable future. In addition, understanding how xenobiotics affect cellular and organismal physiology can provide valuable clues to how normal physiological systems function and how changes in those systems can lead to diseases like diabetes.
